# Supplementary material for: When the liver is in poor condition, so is the heart – cardiac remodelling in MASH mouse models
Source: Clin Sci (Lond). 2024 Sep 17;138(18):1151–71. doi: 10.1042/CS20240833 (PMC11405860; doi:10.1042/CS20240833)
Supplement: Supplementary Figures S1-S2 and Tables S1-S2 [file CS-2024-0833_supp.pdf]

Supplements, table 1: comprehensive dietary compositions. The following information of ingredients were adopted from the manufacturers' information. **Normal diet (SAFE® A03):** wheat, maize, wheat bran, barley, extruded soybeans, soybean meal, hydrolyzed fish proteins, inactivated brewer's yeast, calcium carbonate, pre-mixture of vitamins, pre-mixture of minerals, dicalcium phosphate; no added fat. **High fat diet (D12492):** casein (lactic, 30 Mesh), L-cystine, Lodex 10, sucrose (fine granulated), Solka Floc (FCC200), lard, soybean oil (USP), S10026B (minerals), V10001C (vitamins), choline bitartrate, dye (Blue FD&C #1, Alum. Lake 35-42%). **Western Diet (D05011404):** casein (lactic, 30 Mesh), methionine (DL), sucrose (fine granulated), Lodex 10, starch (corn), Solka Floc (FCC200), butter (anhydrous, GrassLand), corn oil, S10001 (minerals), calcium carbonate (Light, USP), V10001 (vitamins), choline bitartrate, ethoxyquin, cholesterol (NF), dye (Blue FD&C #1, Alum. Lake 35-42%). **Additional kcal in form of fructose (not included in this list) were only contained in the drinking water of mice fed the Western Diet.**

component	Normal diet		High fat diet		Western Diet	
	g / 100g of diet	% kcal	g / 100g of diet	% kcal	g / 100g of diet	% kcal
Carbohydrates, total	52.00	61.3	25.56	20.0	48.88	42.8
simple	4.40	-	9.41	-	33.95	-
complex	33.50	-	16.15	-	14.93	-
Lipids, total	5.10	13.5	34.89	60.0	20.91	40.4
SFAs	0.91	-	12.92	-	12.45	-
MUFAs	1.05	-	15.01	-	5.99	-
PUFAs	2.78	-	5.41	-	1.28	-
Protein	21.40	25.2	26.23	20.0	19.71	16.8
Fibre	4.00	-	6.46	-	4.98	-
Vitamins, total	1.39	-	0.39	-	1.20	-
choline	0.21	-	0.11	-	0.08	-
Minerals, total	3.21	-	6.46	-	3.88	-
Cholesterol	-	-	-	-	0.50	-
Choline (bitartrate)	0.21		0.26		0.20	

Energy (kcal/g)	3.40	5.20	4.54

Supplements, table 2: in vivo evaluation of heart function in WT/Foz mice by echocardiography. Cardiac parameters were examined after 12 (white fields) and 20 (black fields) weeks of feeding. E/A- and E/e'-ratio of FH mice are not measurable (n.m.) in older animals since exaggerated obesity prevented the acquisition of data of adequate validity. FS was analyzed via M-mode, all remaining parameters were measured using B-mode. **Statistics: n=5-6 per group; the focus of this examination was the potential impact of MASH, thus p-values are displayed for comparison of FH (MASH liver) with WN (healthy liver) values; n.s.=not significant; two-way ANOVA (2wA) with Tukey's multiple comparisons test; Kruskal-Wallis (K-W) with Dunn's multiple comparisons test.**

parameter	WN	WH	FN	FH	p-value	statistical test
Body weight (BW), g	26.85 ± 0.54	31.15 ± 0.54	30.53 ± 1.31	55.12 ± 1.22	***	K-W
	28.58 ± 0.93	36.07 ± 0.87	38.44 ± 2.56	63.78 ± 1.49	***	K-W
End-systolic volume (ESV), µL	13.70 ± 1.22	13.52 ± 1.82	11.95 ± 0.73	13.66 ± 1.10	n.s.	K-W
	12.29 ± 1.60	13.51 ± 1.20	15.21 ± 1.33	12.92 ± 1.18	n.s.	K-W
End-diastolic volume (EDV), µL	39.23 ± 2.21	40.66 ± 2.15	39.35 ± 2.26	43.69 ± 2.38	n.s.	K-W
	40.92 ± 3.95	49.40 ± 2.87	45.30 ± 2.99	49.35 ± 2.01	n.s.	K-W
Ejection fraction (EF), %	65.10 ± 2.16	67.39 ± 3.59	69.47 ± 1.39	68.76 ± 1.63	n.s.	K-W
	70.01 ± 2.13	72.83 ± 1.63	66.44 ± 1.44	73.91 ± 2.09	n.s.	K-W
Fractional shortening (FS), %	44.67 ± 3.54	45.43 ± 2.05	44.09 ± 2.23	46.15 ± 2.30	n.s.	K-W
	44.17 ± 2.70	46.44 ± 2.29	46.77 ± 2.71	49.60 ± 1.52	n.s.	K-W
Left ventricular (LV) mass, mg	137.60 ± 1.06	145.7 ± 9.55	144.90 ± 5.11	164.2 ± 9.06	n.s.	2wA
	135.30 ± 11.03	154.30 ± 10.74	154.50 ± 15.65	165.60 ± 6.72	n.s.	2wA
Heart rate (HR), bpm	566.80 ± 11.20	548.60 ± 14.46	533.50 ± 14.50	526.70 ± 20.22	n.s.	2wA
	552.10 ± 12.27	540.30 ± 11.87	548.30 ± 13.45	469.10 ± 34.53	*	2wA
Left ventricular posterior wall at end systole (LVPWs), mm	1.32 ± 0.06	1.39 ± 0.07	1.35 ± 0.06	1.62 ± 0.07	n.s.	K-W
	1.37 ± 0.07	1.43 ± 0.06	1.50 ± 0.06	1.68 ± 0.05	*	K-W
Left ventricular posterior wall at end diastole (LVPWd), mm	0.77 ± 0.03	0.82 ± 0.04	0.83 ± 0.06	0.99 ± 0.05	*	K-W
	0.81 ± 0.03	0.83 ± 0.03	0.88 ± 0.03	0.98 ± 0.04	*	K-W
Interventricular septum at end systole (IVSs), mm	1.23 ± 0.02	1.27 ± 0.06	1.27 ± 0.06	1.49 ± 0.07	*	2wA
	1.24 ± 0.06	1.40 ± 0.08	1.43 ± 0.08	1.59 ± 0.06	*	2wA
Interventricular septum at end diastole (IVSd), mm	0.82 ± 0.02	0.87 ± 0.04	0.84 ± 0.05	1.00 ± 0.06	*	2wA
	0.84 ± 0.05	0.93 ± 0.05	0.91 ± 0.05	1.07 ± 0.08	*	2wA
Left ventricular internal diameter at end-systole (LVIDs), mm	1.86 ± 0.16	1.86 ± 0.10	1.92 ± 0.12	1.95 ± 0.12	n.s.	2wA
	1.87 ± 0.11	1.97 ± 0.12	1.93 ± 0.10	1.93 ± 0.08	n.s.	2wA
Left ventricular internal diameter at end-diastole (LVIDd), mm	3.33 ± 0.08	3.39 ± 0.08	3.42 ± 0.11	3.63 ± 0.14	n.s.	2wA
	3.34 ± 0.07	3.67 ± 0.10	3.64 ± 0.10	3.82 ± 0.10	**	2wA
E/A ratio	2.16 ± 0.26	1.82 ± 0.15	2.01 ± 0.13	1.69 ± 0.23	n.s.	2wA
	2.55 ± 0.23	2.09 ± 0.31	2.84 ± 0.33	n.m.	-	-
E/e' ratio	32.33 ± 0.54	35.19 ± 2.66	27.36 ± 0.61	26.47 ± 6.53	n.s.	K-W
	29.97 ± 1.39	31.37 ± 1.82	29.09 ± 3.12	n.m.	-	-

A.

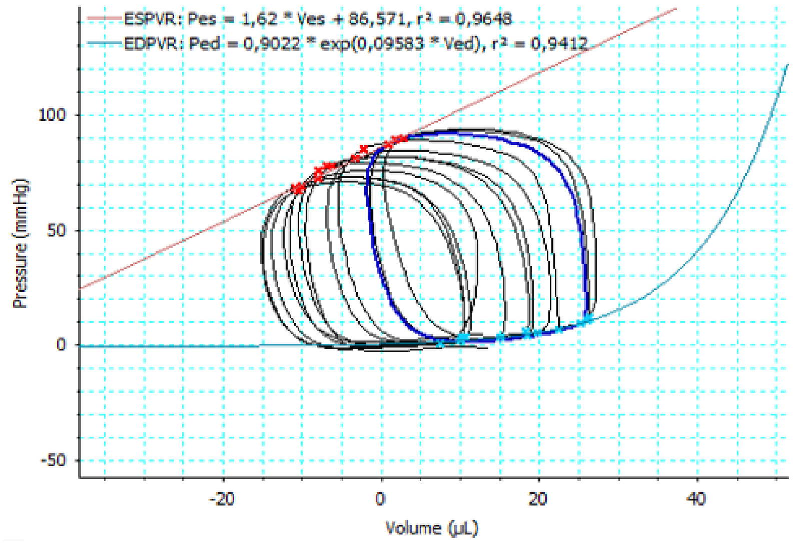

B.

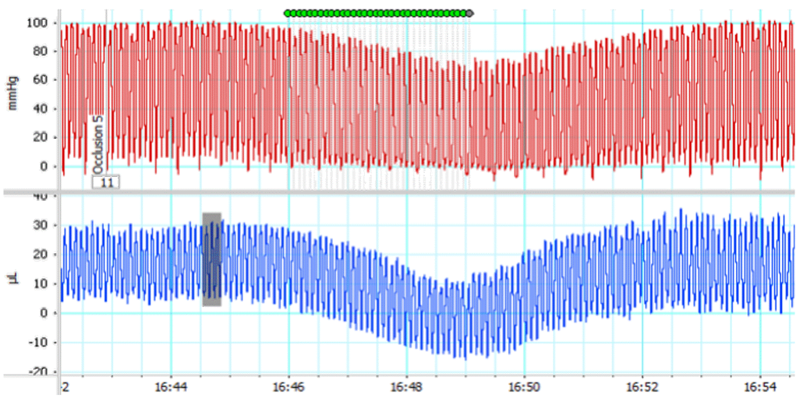

Supplements, figure 1: representative PV loop data recording. (A) PV loops during vena cava occlusion in WN mouse; (B) left ventricular pressure (upper part, red) and volume (lower part, blue) changes.

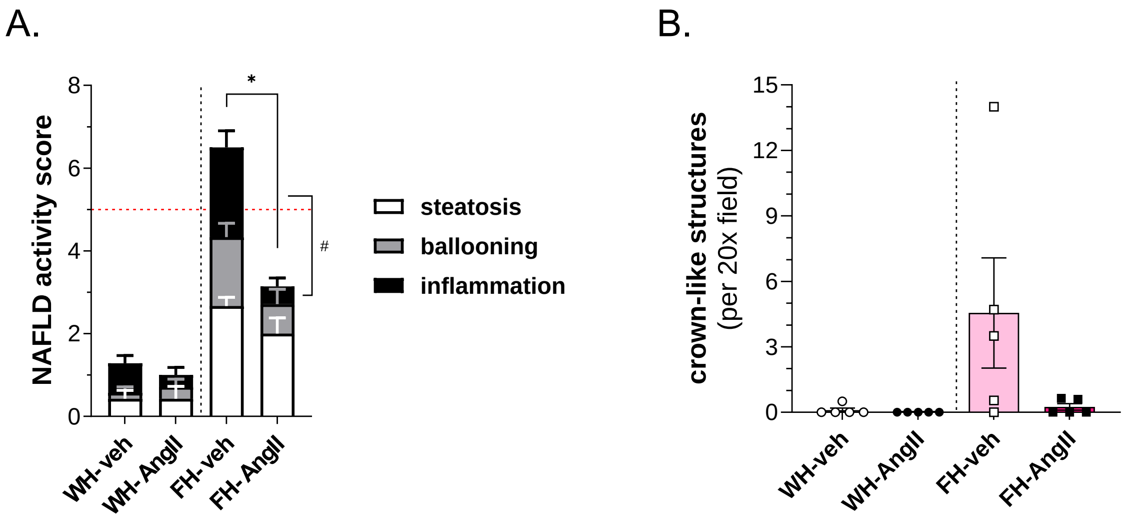

Supplements, figure 2: impact of 4 weeks of AngII-treatment on hepatic conditions in WT/Foz mice on HFD. (A) NAS based on evaluation of steatosis (white bars), ballooning (grey bars) and inflammation (black bars), threshold for MASH displayed as dashed red line; (B) number of crown-like structures per 20x field of view (using F4/80-stained sections). **Animals: n=5-7 per group; Statistics: impact of AngII-treatment compared with vehicle-treatment was performed for Foz and wild type mice separately; (Foz, inflammation): unpaired two-tailed t-test; (all remaining tests, for Foz and WT): unpaired two-tailed Mann-Whitney test.**
